# Immune Effector Cell Associated Neurotoxicity (ICANS) in Pediatric and Young Adult Patients Following Chimeric Antigen Receptor (CAR) T-Cell Therapy: Can We Optimize Early Diagnosis?

**DOI:** 10.3389/fonc.2021.634445

**Published:** 2021-03-08

**Authors:** Brandon Douglas Brown, Francesco Paolo Tambaro, Mira Kohorst, Linda Chi, Kris Michael Mahadeo, Priti Tewari, Demetrios Petropoulos, John M. Slopis, Zsila Sadighi, Sajad Khazal

**Affiliations:** ^1^ Department of Pediatrics, Pediatric Stem Cell Transplantation and Cellular Therapy, The University of Texas MD Anderson Cancer Center, Children’s Cancer Hospital, Houston, TX, United States; ^2^ Bone Marrow Transplant Unit, Pediatric Oncology Department, AORN Santobono Pausilipon, Naples, Italy; ^3^ Division of Pediatric Hematology/Oncology, Department of Pediatric and Adolescent Medicine, Mayo Clinic, Rochester, MN, United States; ^4^ Division of Diagnostic Imaging, Department of Neuroradiology, The University of Texas MD Anderson Cancer Center, Houston, TX, United States; ^5^ CARTOX Program, The University of Texas MD Anderson Cancer Center, Houston, TX, United States; ^6^ Department of Pediatrics, Neuro-Oncology/Neurology, Children’s Cancer Hospital, The University of Texas at MD Anderson Cancer Center, Houston, TX, United States

**Keywords:** immune effector cell, immune effector cell neurotoxicity, Cornell Assessment for Pediatric Delirium, immune effector cell-associated neurotoxicity, delirium

## Abstract

The Cornell Assessment for Pediatric Delirium (CAPD) was first proposed by the Pediatric Acute Lung Injury and Sepsis Investigators Network-Stem Cell Transplantation and Cancer Immunotherapy Subgroup and MD Anderson CARTOX joint working committees, for detection of immune effector cell associated neurotoxicity (ICANS) in pediatric patients receiving chimeric antigen receptor (CAR) T-cell therapy. It was subsequently adopted by the American Society for Transplantation and Cellular Therapy. The utility of CAPD as a screening tool for early diagnosis of ICANS has not been fully characterized. We conducted a retrospective study of pediatric and young adult patients (n=15) receiving standard-of-care CAR T-cell products. Cytokine release syndrome (CRS) and ICANS occurred in 87% and 40% of patients, respectively. ICANS was associated with significantly higher peaks of serum ferritin. A change in CAPD from a prior baseline was noted in 60% of patients with ICANS, 24–72 h prior to diagnosis of ICANS. The median change from baseline to maximum CAPD score of patients who developed ICANS versus those who did not was 13 versus 3, respectively (p=0.0004). Changes in CAPD score from baseline may be the earliest indicator of ICANS among pediatric and young adult patients which may warrant closer monitoring, with more frequent CAPD assessments.

## Introduction

Advances in cellular and immunotherapies such as chimeric antigen receptor (CAR) T-cells used in conjunction with lymphodepleting conditioning regimens are associated with high response rates in hematologic malignancies. Among patients with relapsed or refractory acute lymphoblastic leukemia, overall remission rates of almost 80% at 6 months and durable remissions up to 24 months have been observed (1). CARs are synthetic receptors which can be engineered ex-vivo to human T-cells, reprogramming them to acquire anti-tumor properties. Anti-CD19 CAR T-cells are genetically programmed to bind CD19 antigen present on tumor cells in B-cell hematologic malignancies and induce apoptosis (2). Three CAR T-cell products have been approved by the Food and Drug Administration (FDA) in the United States for the treatment of pediatric, adolescent and young adult patients with relapsed or refractory B-cell ALL and adults with large B-cell and mantle cell lymphomas. These second-generation CAR T-cells utilize a CD3ζ signaling domain with either a CD28 or 4-1BB (CD137) co-stimulatory domain to promote T-cell activation and proliferation (3). Binding of CD19 on target tumor cells activates anti-CD19 CAR T-cells independent of major histocompatibility complex (MHC) with down-stream intracellular signaling ultimately leading to cytokine secretion, proliferation, and lytic activity (4).

CAR T-cell therapy may be associated with unique and potentially life-threatening adverse effects, such as cytokine release syndrome (CRS) and immune effector cell-associated neurotoxicity (ICANS) (2, 5, 6). CRS is an acute systemic inflammatory syndrome that may occur following CAR infusion. It is characterized by fever, hypoxia and hypotension. Activated CAR T-cells release pro-inflammatory cytokines including IL-2, IL-6, and IFNγ leading to T lymphocyte, monocyte and macrophage activation further perpetuating a supraphysiologic release of IL-1RA, IL-10, IL-6, IL-8, IFNα, and other cytokines and chemokines (7). ICANS may occur concomitantly with CRS, following resolution of CRS, or in the absence of CRS (8). Shalabi et al. have prospectively assessed neurotoxicity associated with anti-CD22 CART in a phase 1 clinical trial. Their evaluation included lumbar punctures, brain MRI, cognitive evaluations and a CART specific neurologic symptom checklist (NSC) that was completed by the caregivers prior to cell infusion and repeated post-infusion at approximately day 14 and day 21–28. Significant increase in neurologic symptoms were observed around the time of CART expansion with a significant decrease in symptoms at the final evaluation, consistent with the general reversibility of CART related neurotoxicity (9). Acute symptoms of ICANS may range from subtle inattention, dysgraphia, language disturbance, confusion/altered mental status (AMS), and may progress to seizures or cerebral edema. Prompt diagnosis and appropriate management of ICANS remains imperative as it may otherwise lead to rapid clinical deterioration, warranting acute intervention (10). The precise underlying pathophysiology of ICANS remains poorly understood. Autopsy and animal model data suggest this may be a disorder of the neurovascular unit related to cytokine-mediated endothelial activation and compromise of the blood-brain barrier leading to extravasation of inflammatory cytokines into cerebrospinal fluid (CSF) and brain parenchyma (11).

Although the acute symptoms of toxicities such as CRS and ICANS are generally reversible, they are associated with significant morbidity and up to 47% of patients may require admission to an intensive care unit (ICU) (12). Joint recommendations from the Pediatric Acute Lung Injury and Sepsis Investigators (PALISI) Network-Stem Cell Transplantation and Cancer Immunotherapy (SCT-CI) Subgroup and MD Anderson CARTOX working committees were developed to diagnose and manage these complications in younger patients, improve safety, and standardize monitoring (10). Subsequently, the American Society for Transplantation and Cellular Therapy (ASTCT) developed consensus criteria for diagnosis and grading of CRS and ICANS in adult and pediatric patients receiving immune effector cell (IEC) therapies such as CAR T-cells (13). Key elements of the PALISI-CARTOX joint recommendations were adopted by the ASTCT, including the use of the Cornell Assessment for Pediatric Delirium in the assessment of ICANS in pediatric patients (10, 13, 14).

In adults, the 10-point Immune Effector Cell-Associated Encephalopathy (ICE) score (**Supplementary Figure 1**) is used for the grading of ICANS together with other neurologic domains, such as level of consciousness, motor symptoms, seizures, and signs of elevated ICP/cerebral edema (13). The ICE score was adopted by the ASTCT as a modification of the CARTOX-10 score and consists of assessments regarding orientation, naming, ability to follow commands, writing and attention (7, 13). Dysgraphia, in particular, has been noted to be an early indicator of ICANS in adult patients (7). Although the 10-point ICE screening tool provides a reliable, objective grading system for adults, the tool is inappropriate for children particularly for those younger than 12-years of age or among developmentally delayed patients. The Cornell Assessment of Pediatric Delirium (CAPD) was developed and validated as a rapid screening tool for delirium in pediatric intensive care units (**Supplementary Figure 2**). The tool has been validated for up to 21 years of age and developmental anchor points may be used to ensure appropriate assessments (14). Recently, CAPD has been suggested for potential use in adolescent & young adult patients up to 25 years of age (15). CAPD is specifically used in lieu of the ICE score for ICANS assessment in children (13).

To date, the impact of CAPD as a screening tool for early recognition of ICANS in patients receiving CART therapies has not been fully described. Although a CAPD score < 9 does not signify delirium present, we hypothesized that a rise in CAPD scores in patients from their clinical baseline may be associated with increased risk for ICANS.

## Materials and Methods

We conducted a retrospective study of all patients receiving standard-of-care CAR T-cell products, aged 25 years or younger treated at the Children’s Cancer Hospital at MD Anderson Cancer Center, from March 2018 through April 2020. This study was approved by our institutional review board. Data were collected from electronic medical records. All patients receiving standard of care CAR T-cell therapies were admitted to our inpatient unit for lymphodepletion until a minimum of day + 7 following infusion per our standard guidelines during the study period. Supportive care and monitoring for development of CRS and ICANS were performed based on institutional guidelines as previously described (prior to adoption of ASTCT guidelines) (10).

During phased implementation of ASTCT guidelines, patients had assessments performed which fulfilled both our pre-existing institutional and ASTCT guidelines. Therefore, CAPD was assessed in patients receiving CAR T-cell therapy (up to age 25 years), as was a hand-writing sample when possible (including patients < 12 years), beginning at admission prior to lymphodepletion. As CAPD has only been validated in patients up to 21 years and is recommended by ASTCT for use in patients younger than 12 years, the CARTOX-10 score was assessed for patients ≥ 12 years of age, if developmentally appropriate. During phased implementation of the ASTCT grading, ICE scores replaced CARTOX-10 assessments. ICE and CARTOX-10 scores were considered equivalent for the purpose of this study which is focused on CAPD. Both CAPD and ICE scores, where applicable, were assessed and recorded at minimum twice per day (beginning on the day of admission for lymphodepletion) by the patient’s assigned nurse at each end-of-shift which were reviewed and confirmed by the treating physician daily.

We retrospectively re-graded patients, where necessary, based on prior documentation in accordance with current ASTCT age-appropriate ICANS grading recommendations (**Supplementary Figure 3**) (13). There is no Common Terminology Criteria for Adverse Events Version 5.0 (CTCAE v5) specific for ICANS or neurotoxicity related to cellular therapy. Generalized CTCAE neurologic adverse events (AE) were also retrospectively assessed based on the maximum clinical neurological sign or symptom documented (16). All neurological data including clinical presentation, ICANS scoring, electroencephalography (EEG), CSF and magnetic resonance imaging (MRI) data were reviewed by a board-certified neurologist while the MRI also reviewed by a board-certified neuroradiologist. All patients were started on levetiracetam for anti-epileptic prophylaxis beginning after admission prior to cell infusion and continued for a minimum of 30 days after cell infusion.

In this study, a patient’s baseline CAPD score is defined as the mean of the first 2 CAPD scores in the first 24 h of admission for lymphodepletion prior to CAR T-cell infusion. Delta CAPD is defined as the change from this baseline to maximum CAPD score. Descriptive statistics were used to summarize the demographics of this retrospective sample. Two tailed, two samples equal variance t-tests were used to compare the differences in delta CAPD scores and serum biomarkers.

## Results

### Demographics

Demographics are summarized in **Table 1**. A total of 15 patients received standard of care CAR T-cell therapy during the study period. Underlying diagnosis was pre-B cell ALL in the majority (n=14) of patients (received tisagenlecleucel) and primary mediastinal large B cell lymphoma (n=1; received axicabtagene ciloleucel).

**Table 1:** Patient demographics and details of toxicities including CRS and ICANS.

Pt	Age (years)	Gender	Primary Diagnosis	Disease Burden	CART Product	Max. CRS Grade	Max. ICANS Grade	Symptoms	CRS Onset from Infusion & Treatment	ICANS Onset from Infusion & Treatment	Baseline CAPD	First Increase CAPD prior to ICANS	Max.CAPD prior to ICANS	Max. CAPD	Min. ICE	CTCAE V5 Neurotoxicity	ICANS Duration
1	23	M	Pre-B ALL, BM relapse	PLD PB: WBC 13.5 K/μl, Blasts 14%	Tisagen-lecleucel	3	2	AMS, dysgraphia	7 days; tocilizumab × 4 doses	9 days; dexamethasone	0	48 h	4	N/A	3	Grade 2: Moderate disorientation and dysphasia	< 24 h
2	8	F	Pre-B ALL, BM and CNS relapse	PLD BM: Blasts 30%, MRD 20%	Tisagen-lecleucel	3	3	AMS, aphasia, dysgraphia	6 days	7 days; dexamethasone	1	72 h	10	14	–	Grade 3: Severe disorientation and dysphasia	96 h
3	18	F	Primary Mediastinal Large B-cell Lymphoma	PLP PETCT: Disease progression, Deauville score 5	Axicabta-gene Ciloleucel	1	3	AMS, dysgraphia	6 days; tocilizumab × 1 dose	6 days; dexamethasone	1	0 h	0	14	1	Grade 3: Severe disorientation and dysphasia	24 h
4	9	M	Pre-B ALL, BM and CNS relapse	PLD PB: WBC 4.2 K/μl, Blasts 1% PLP BM: MRD 96%	Tisagen-lecleucel	4	3	GTC Seizure, AMS, dysgraphia	2 days; tocilizumab × 3 doses, siltuximab	12 days; dexamethasone	1	48 h	4	5	–	Grade 2: Seizure	< 24 h
5	22	F	Pre-B ALL, breast relapse	PLP BM: Blasts 3%, MRD negative	Tisagen-lecleucel	1	3	AMS, aphasia	3 days; tocilizumab × 1 dose	5 days	0	0 h	0	7	0	Grade 3: Severe somnolence	< 24 h
6	25	F	Pre-B ALL, BM and CNS relapse	PLD PB: WBC 9.4 K/μl, Blasts 37%	Tisagen-lecleucel	3	3	AMS, dysgraphia	7 days; tocilizumab × 4 doses	8 days; dexamethasone	0	24 h	10	16	1	Grade 3: Severe disorientation and dysphasia	168 h

Baseline CAPD assigned is the mean of the first two CAPD scores after admission prior to cell infusion. CRS and ICANS grades correspond to current ASTCT consensus grading guidelines. Resolution of ICANS corresponded to return of CAPD score to patient’s baseline and resolution of symptoms as documented by clinical assessments. Patient 1 had serial ICE scores and did not have CAPD scores available around the time of ICANS diagnosis.

ALL, acute lymphoblastic leukemia; PB, peripheral blood; BM, bone marrow; CNS, central nervous system; PLP, pre-leukapheresis; PLD, pre-lymphodepletion; WBC, white blood cell; AMS, altered mental status; GTC, generalized tonic-clonic; CTCAE, Common Terminology Criteria for Adverse Events.

### CRS/ICANS

Cytokine release syndrome (CRS), median CRS grade 2 (range 1–4), was observed in 13 of 15 (87%) patients with onset at a median of 6 days post CAR T-cell infusion (range 2–11). Immune effector cell-associated neurotoxicity syndrome (ICANS), median ICANS grade 3 (range 2–3), occurred in six of 15 (40%) patients with onset at a median of 8 days post CAR T-cell infusion (range 5–12 days). All cases of ICANS occurred concurrently with CRS at a median time of 2 days following onset of CRS (range 0–10 days). No patients developed late onset or recurrent ICANS. No significant differences in age (*p=0.7*), gender (*p=0.6*), product type (*p=0.4*), or presence of CNS disease (*p=0.32*) between patients who developed ICANS and those who did not were detected.

### CAPD and ICANS Screening

Baseline CAPD was assessed in all patients and serial CAPD assessments were available for 14 patients. One patient over 21 years of age, had serial ICE but did not have consistent CAPD assessments performed. The CARTOX-10/ICE score was calculated for patients ≥ 12 years of age if developmentally appropriate (n=11). Baseline CAPD scores ranged between 0 and 2 for most patients (n=14); a patient with Trisomy 21 had baseline CAPD score ranging from 2 to 4. No change in baseline CAPD was detected within 48 h of starting levetiracetam for any patient in our cohort. As expected, we observed a wider variation in CAPD score from baseline (delta CAPD) in patients who developed ICANS than those who did not. The median change from baseline to maximum CAPD of patients who developed ICANS versus those who did not was 13 (n=5, range 4–16) versus 3 (n=9, range 1–4), respectively (p=0.0004). Among evaluable patients who developed ICANS, the median peak CAPD score at diagnosis was 14 (n=5, range 5–16). All patients >18 years old with ICANS (n=4) had baseline ICE scores of 10 and median ICE score of 1 (range 0–3) at the time of diagnosis of ICANS.

**Figure 1:**
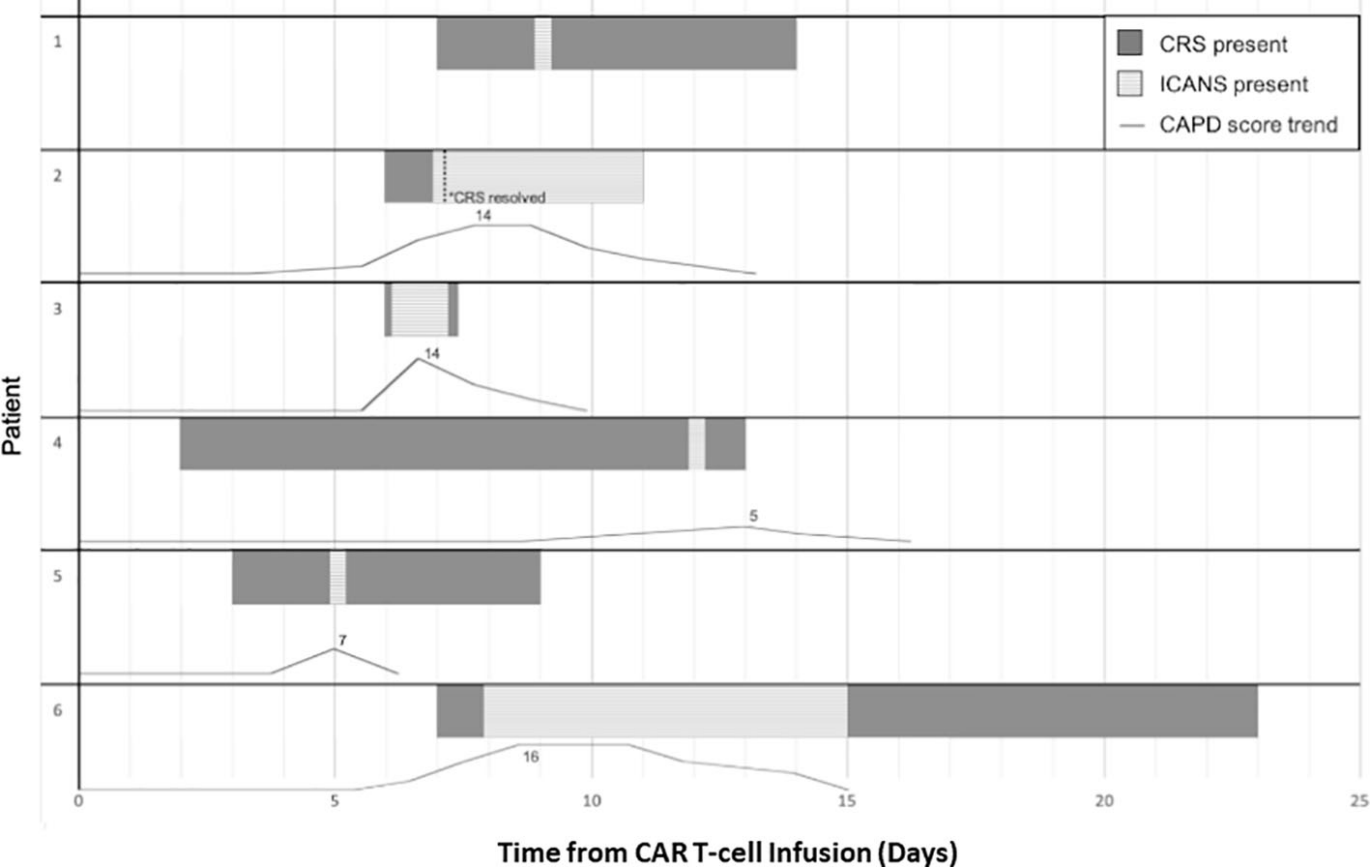
Timeline in days of toxicity, CRS and ICANS, onset and offset for patients that developed ICANS with corresponding trend of CAPD scores with day 0 representing day of cell infusion. Patient numbers corresponding to **Table 1**. Patient 1 had serial ICE scores and did not have CAPD scores available around the time of ICANS diagnosis. Patient 6 developed ICANS which slowly returned to baseline over the course of seven days.

In the five patients that developed ICANS with CAPD scores available for assessment, three developed changes in CAPD scores above their baseline, a minimum of 24 h prior to ICANS diagnosis (range 24–72 h), with a mean rise in CAPD score by seven points (range 3–10) 24 h prior to diagnosis. Two of these patients (**Table 1**, Patients 2 and 6) had an initial increase in CAPD > 9, with delirium that was initially attributed to causes other than ICANS (pain medication). Both later developed ICANS with further increase in CAPD scores and other overt manifestations of ICANS. The remaining patients (n=2) had simultaneous occurrence of first CAPD change above baseline, maximum CAPD, and diagnosis of ICANS. For the patients under 12 years of age (n=3), two developed ICANS and both showed an initial change above baseline 72 h and 48 h prior to ICANS diagnosis, respectively (**Figure 1**).

Two out of six patients developed aphasia at time of ICANS and all six patients had altered mental status or decreased level of consciousness. Patient 6 developed ICANS with waxing and waning confusion associated with CAPD scores that slowly returned to baseline over the course of seven days (**Figure 1**). As outlined in **Table 1**, two patients developed ICANS with acute events including seizure and depressed level of consciousness without detectable preceding delirium. Patient 4 (**Table 1**) developed a brief (2 min), generalized tonic-clonic (GTC) seizure (grade 3 ICANS) 10 days following onset of CRS. This patient had been admitted to ICU prior to CART infusion for acute renal failure (tumor lysis syndrome) requiring continuous renal replacement therapy. He then developed grade 4 CRS (with four episodes of pulseless electrical activity requiring cardiopulmonary resuscitation) and received anti-cytokine therapy per our institutional guidelines at that time (10). At ICANS diagnosis, computerized tomography (CT) of the brain did not show any acute changes. EEG demonstrated generalized slowing without epileptiform discharges. Patient 5 (**Table 1**) had a self-limited brief episode of altered mental status and aphasia that was identified by an abnormal ICE score at that time, with a change in CAPD score from baseline, (though this score remained <9 as she was appropriately scored based on her status over a shift period and not solely based on that individual timepoint).

Five out of the six patients (83%) who developed ICANS had changes in handwriting (either new inability to write (n=4) or abnormal writing sample (n=1). For the patients who were under 12 years of age, both were acutely unable to produce a writing sample (delirium present). In all patients, handwriting changes were noted either in the immediate period (within 12 h) prior to or at the time of ICANS diagnosis. As shown in **Table 1**, CTCAE v5 grade based on the maximum clinical neurological signs or symptoms observed, corresponded to comparable maximum ICANS grades in our cohort.

### Imaging, Electroencephalograms, and Lumbar Punctures

Baseline brain MRI was obtained in all patients prior to cell infusion. Several (n=4) patients in both groups, (with/out ICANS), were noted to have subtle diffuse periventricular white matter (PVWM) changes on T2/FLAIR sequences (**Table 2**). Subsequent to developing ICANS, neuroimaging (MRI and/or CT) was obtained in all patients with ICANS (n=6) with unremarkable findings. As delineated in **Tables 1** and **2**, patient 1 had baseline PVWM changes of higher intensity than would be expected for patient age as well as progression of non-specific, diffuse PVWM changes after developing ICANS. Patient 4 had stable, small bilateral subdural hematomas unchanged from baseline. Two patients that did not develop ICANS also demonstrated progression of non-specific, diffuse PVWM changes. EEG was not obtained at baseline routinely, however was obtained in 5 affected patients at the time of ICANS which all (n=5) demonstrated generalized slowing and diffuse disturbance of cerebral activity and 1 with additional frontal intermittent rhythmic delta activity (FIRDA) or frontal slowing (**Table 2**). In the remaining patient, EEG electrode placement was unable to be tolerated due to agitation.

**Table 2:** Neuroimaging and evaluation study findings in patients with ICANS.

Pt	Baseline MRI Findings	MRI Findings at time of ICANS	EEG Findings at time of ICANS
1	Pineal cyst, PVWM changes higher intensity than expected for age	Unchanged pineal cyst, mild progression of PVWM changes	Diffuse slowing
2	PVWM changes	Unchanged PVWM changes	Not available
3	Unremarkable	Unremarkable	Diffuse slowing with prominent FIRDA
4	Bilateral SDH	Stable bilateral SDH	Diffuse slowing
5	Unremarkable	Unremarkable	Diffuse slowing
6	Unremarkable	Unremarkable	Diffuse slowing

Baseline MRIs for this purpose are the most recent available study prior to CAR T-cell infusion.

MRI, magnetic resonance imaging; EEG, electroencephalogram; PVWM, periventricular white matter; SDH, subdural hematoma; FIRDA, frontal intermittent rhythmic delta activity.

Lumbar puncture was not performed at the time of ICANS in any patient (n=6). Lumbar puncture was performed in a single patient (**Table 1**, Patient 6) with history of ICANS following discharge after presenting emergently with altered mental status 5 weeks after CAR T-cell infusion and 3 weeks after resolution of ICANS. Cerebrospinal fluid demonstrated sterile lymphocytosis without evidence of blast cells. The patient was subsequently diagnosed with acute multi-substance intoxication.

### Serum Biomarkers

Baseline c-reactive protein (CRP) and ferritin prior to CAR T-cell infusion as well as peak levels derived from serial measurements were obtained in all patients (n=15). All patients developed marked elevations in both CRP and ferritin. The average peak CRP (mg/L) in patients with ICANS compared to patients that did not develop ICANS were 190 mg/L (n=5, range 94-300) and 173 mg/L (n=9, range 74-300), respectively (*p=0.7*). The average peak ferritin (ng/mL) in patients with ICANS compared to those without were 56,010 (n=6, range 1,905–128,000) and 10,640 (n=9, range 1,075–44,000), respectively (*p=0.04*). Pro-inflammatory cytokines interleukin 6 (IL-6), tumor necrosis factor α (TNFα), and interferon γ (IFNγ) were assessed at baseline prior to CAR T-cell infusion in 12 patients and peak levels derived from serial measurements were obtained in 13 patients. The average peak IL-6 (pg/mL) in patients with ICANS compared to patients that did not develop ICANS were 49,560 (n=4, 157–181,000) and 1,890 (n=9, range 14–10,660), respectively, although this did not meet statistical significance (*p=0.11*). Similarly, the mean peak IFNγ (pg/mL) in patients with ICANS compared to patients without were 35,160 (n=4, range 14–113,300) and 700 (n=9, range 0–5,696), respectively, although this did not meet statistical significance (*p=0.07*).

### Intensive Care Support

All patients with ICANS required transfer to ICU. Two were specifically transferred to the ICU for ICANS monitoring but did not require any critical care interventions. The other patients developed ICANS while in ICU (n=2) for management of grade 3 CRS (hypoxia n=1, hypotension n=1) or developed ICANS while in ICU (n=2) having been admitted there prior to CART cell infusion for renal failure and respiratory failure, respectively. The remaining patients without ICANS (n=9) did not require intensive care (n=5) or required intensive care (n=4) for management of CRS (n=2), for management of complications related to primary disease after failed CART-cell therapy (n=1) or were in ICU prior to CAR T-cell infusion for respiratory failure (n=1).

### Treatment

Of the six patients with ICANS, five received the IL-6 receptor monoclonal antibody, tocilizumab, for concurrent CRS. Tocilizumab was administered prior to the development of ICANS in three patients for the treatment of CRS. Tocilizumab was administered at the onset of ICANS in a total of two patients with concurrent grade 1 CRS after 12-h and 48-h of fever, respectively. Dexamethasone was administered (total n=6) for specific management of ICANS.

### Primary Disease Assessment and Long-Term Follow-Up

All patients had disease assessment around 30 days post CAR T-cell infusion. Of patients with pre-B ALL (n=14), nine patients were MRD negative and one patient had morphologic remission with incomplete hematologic recovery. The remaining patients had confirmed refractory leukemia on bone marrow biopsy (n=3) and/or circulating peripheral blasts (n=1). Of the six patients with ICANS, only one did not respond to CAR T-cell therapy with 30-day evaluation demonstrating 68% blasts in bone marrow. At a median follow-up of 9 months (range 3–28 months), we found no significant differences in ICU survival, 30 day remission rates and/or relapse free survival and/or overall survival and/or performance scores (Lansky/Karnofsky) between patients that developed ICANS and those that did not.

## Discussion

Among adult patients receiving IEC therapies, the ICE score is used to assess mental status and promptly detect ICANS, but this may not be appropriate for younger children and those with developmental delay. The CAPD screening tool may be used in children to detect delirium, which is present in more than 25% of patients admitted to pediatric intensive care units (17–19). Delirium is an acute syndrome with fluctuating awareness and cognition, and it may be an early indicator of ICANS. In general, children diagnosed with delirium have increased short- and long-term morbidity, and sometimes excess mortality, but the extent to which this is true among patients who develop ICANS remains to be clearly defined (20–22).

The CAPD assesses pediatric delirium within the context of child development by comparing the detection of specific items on a scale addressing consciousness, cognition, orientation, psychomotor activity, and affect/distress with anchor points that characterize the development of the patient by age groups (23, 24). CAPD has been validated as a tool for detection of delirium in critically ill and/or younger oncology patients (14, 25, 26). The tool has also been successfully implemented across different cultures and in different languages (27–29). An earlier report, suggests that the CAPD is a sensitive screen for ICANS among patients with relapsed or refractory B‐ALL who were enrolled in phase I of an open label phase I/II clinical trial (clinicaltrials.gov NCT02028455) of CD19‐directed, 4‐1BB co-stimulated CAR T-cells (SCRI‐CAR19v1) (30).

CAPD may be a useful tool for the early detection of delirium and even ICANS in patients receiving CAR T-cells. This may be particularly evident in patients less than 12 years of age as in our cohort, CAPD was noted above baseline at 48 and 72 h, respectively, prior to the diagnosis of ICANS. Among those with a change in CAPD, more frequent assessments may be indicated. As CAPD is an assessment of delirium based on summary observations over time, the intervals between CAPD assessments may be guided by changes in clinical status.

While delirium is an early indicator of ICANS in patients receiving CAR therapies, it is important to consider the differential diagnosis. Patients who have received CAR therapies and develop fever and hypotension may have CRS, but it is also important to rule out infection and when appropriate, initiate empiric sepsis anti-microbial coverage. Similarly, among CART patients with a CAPD score >9, it is important to consider the contribution of other factors such as sepsis, benzodiazepines, opioids, anticholinergics and poor sleep hygiene (26). While independent associations between steroid administration and development of delirium have been described, in the setting of ICANS, steroids are an important abortive treatment (31). For patients that have not been placed on prophylactic seizure medications, a rise in CAPD could serve as an indicator to begin. While prospective studies are needed to elucidate whether delirium from other causes and a rising CAPD from baseline may increase the risk of ICANS, a CAPD score > 9 warrants more frequent monitoring and interventions to reverse delirium.

Although there has been evidence reported for the utility of baseline biomarkers levels as predictors of ICANS, we were unable to detect predictive biomarkers among those tested in our cohort. Biomarkers such as ApoA1 and Angiogenin levels at baseline, and changes in ApoA1 or Angiogenin over time, may indicate risk of ICANS (32). Dysgraphia appears to be a specific finding among patients with ICANS, but changes in CAPD were the earliest indicator of ICANS in our cohort. Pediatric ICU outcomes did not differ among patients with/out ICANS. Despite relatively high critical care admission rates, overall resource utilization studies among adult CAR T patients suggest that organ failure scores should not guide decisions about limiting treatment. Rather, reversibility of the underlying pathology may be the most important factor for survival (33). This may also be true for patients with ICANS in the PICU. In our cohort, while one patient had brief generalized tonic clonic seizure, on EEGs obtained at time of ICANS, diffuse disturbance of cortical activity demonstrated as generalized slowing was a consistent finding in all patients and one additionally with frontal lobe slowing or Intermittent Rhythmic Delta Activity (FIRDA). Seizures and other non-epileptiform activity seen on EEGs including both diffuse slowing and FIRDA have been reported as known findings at the time of ICANS (13). While follow-up EEG is not considered essential when patients improve clinically, it may be a useful practice to follow EEG findings for resolution, as we seek to better understand the long-term outcomes of patients with ICANS. Neuroimaging at baseline and on long-term follow-up may also improve our understanding of the significance and natural course of observed PVWM changes in some patients.

Our study is limited by the retrospective design as well as the overall small sample size of 15 having received CAR T-cell therapy, with 9 pediatric patients (aged 18 years and younger) and only three pediatric patients in the ICANS cohort. Additionally, CAPD has been validated as a delirium screening tool in patients up to the age of 21 years; however, it is analyzed here in young adult patients up to age 25 years which further limit the strength of our findings. Differentiating the specific causes of delirium (medication versus ICANS for example), that may be present in patients receiving CART therapy, was outside the scope of this study, but warrants further investigation.

In summary, a rising CAPD score may represent the earliest indicator of ICANS in pediatric and young adult patients receiving standard of care CD19-directed CAR T-cell therapy and may be an indication for more frequent assessments and closer monitoring. These findings, including the utility of CAPD in patients aged 21 to 25 years, warrant validation in larger prospective cohorts.

## Data Availability Statement

The original contributions presented in the study are included in the article/**Supplementary Material**. Further inquiries can be directed to the corresponding author.

## Ethics Statement

The studies involving human participants were reviewed and approved by Institutional Review Board at The University of Texas MD Anderson Cancer Center. Written informed consent from the participants’ legal guardian/next of kin was not required to participate in this study in accordance with the national legislation and the institutional requirements.

## Author Contributions

SK obtained IRB approval for this study. ZS developed the concept. BB, ZS, MK, FT, and KM collected and analyzed data. LC and ZS reviewed all neuroimaging. ZS reviewed all EEGs. LC, PT, DP, SK, JS, ZS, and KM treated the patients. BB, MK, KM, and SK wrote the manuscript. All authors contributed to the article and approved the submitted version.

## Conflict of Interest

The authors declare that the research was conducted in the absence of any commercial or financial relationships that could be construed as a potential conflict of interest.

## Supplementary Material

Supplementary Figure 1 Immune-effector cell-associated encephalopathy assessment tool for the grading of ICANS. A score of 10 represents no impairment, 7–9 grade 1 ICANS, 3–6 grade 2 ICANS, and 0–2 grade 3 ICANS. A score of 0 due to patient being unarousable and unable to perform assessment corresponds to grade 4 ICANS.

Supplementary Figure 2 Cornell Assessment of Pediatric Delirium. Encephalopathy assessment tool for children < 12 years of age adapted by ASTCT. Scores between 1-8 may represent no impairment, grade 1 or grade 2 ICANS and must be combined with clinical assessment. Score > 8 corresponds to grade 3 ICANS. If unable to perform CAPD due to patient being unarousable, corresponds to grade 4 ICANS.

Supplementary Figure 3 ASTCT ICANS Consensus Grading. ICANS grade is determined by the most severe event (ICE or CAPD score, level of consciousness, seizures, motor findings, raised ICP/cerebral edema) not attributable to any other cause.

## References

[1] Maude SL Frey N Shaw PA Aplenc R Barrett DM Bunin NJ. Chimeric antigen receptor T cells for sustained remissions in leukemia. N Engl J Med (2014) 371(16):1507–17. 10.1056/NEJMoa1407222 PMC4267531 25317870

[2] Shank BR Do B Sevin A Chen SE Neelapu SS Horowitz SB. Chimeric Antigen Receptor T Cells in Hematologic Malignancies. Pharmacotherapy (2017) 37(3):334–45. 10.1002/phar.1900 28079265

[3] Boyiadzis MM Dhodapkar MV Brentjens RJ Kochenderfer JN Neelapu SS Maus MV. Chimeric antigen receptor (CAR) T therapies for the treatment of hematologic malignancies: clinical perspective and significance. J Immunother Cancer (2018) 6(1):137. 10.1186/s40425-018-0460-5 30514386 PMC6278156

[4] Terakura S Yamamoto TN Gardner RA Turtle CJ Jensen MC Riddell SR. Generation of CD19-chimeric antigen receptor modified CD8+ T cells derived from virus-specific central memory T cells. Blood (2012) 119(1):72–82. 10.1182/blood-2011-07-366419 22031866 PMC3251238

[5] Torre M Solomon IH Sutherland CL Nikiforow S DeAngelo DJ Stone RM. Neuropathology of a Case With Fatal CAR T-Cell-Associated Cerebral Edema. J Neuropathol Exp Neurol (2018) 77(10):877–82. 10.1093/jnen/nly064 30060228

[6] Turtle CJ Hanafi LA Berger C Gooley TA Cherian S Hudecek M. CD19 CAR-T cells of defined CD4+:CD8+ composition in adult B cell ALL patients. J Clin Invest (2016) 126(6):2123–38. 10.1172/JCI85309 PMC4887159 27111235

[7] Neelapu SS Tummala S Kebriaei P Wierda W Gutierrez C Locke FL. Chimeric antigen receptor T-cell therapy - assessment and management of toxicities. Nat Rev Clin Oncol (2018) 15(1):47–62. 10.1038/nrclinonc.2017.148 28925994 PMC6733403

[8] Rivera AM May S Lei M Qualls S Bushey K Rubin DB. CAR T-Cell-Associated Neurotoxicity: Current Management and Emerging Treatment Strategies. Crit Care Nurs Q (2020) 43(2):191–204. 10.1097/CNQ.0000000000000302 32084062

[9] Shalabi H Wolters PL Martin S Toledo-Tamula MA Roderick MC Struemph K. Systematic Evaluation of Neurotoxicity in Children and Young Adults Undergoing CD22 Chimeric Antigen Receptor T-Cell Therapy. J Immunother (2018) 41(7):350–8. 10.1097/CJI.0000000000000241 PMC6086728 30048343

[10] Mahadeo KM Khazal SJ Abdel-Azim H Fitzgerald JC Taraseviciute A Bollard CM. Management guidelines for paediatric patients receiving chimeric antigen receptor T cell therapy. Nat Rev Clin Oncol (2019) 16(1):45–63. 10.1038/s41571-018-0075-2 30082906 PMC7096894

[11] Gust J Hay KA Hanafi LA Li D Myerson D Gonzalez-Cuyar LF. Endothelial Activation and Blood-Brain Barrier Disruption in Neurotoxicity after Adoptive Immunotherapy with CD19 CAR-T Cells. Cancer Discov (2017) 7(12):1404–19. 10.1158/2159-8290.CD-17-0698 PMC5718945 29025771

[12] Gutierrez C Brown ART Herr MM Kadri SS Hill B Rajendram P. The chimeric antigen receptor-intensive care unit (CAR-ICU) initiative: Surveying intensive care unit practices in the management of CAR T-cell associated toxicities. J Crit Care (2020) 58:58–64. 10.1016/j.jcrc.2020.04.008 32361219 PMC7321897

[13] Lee DW Santomasso BD Locke FL Ghobadi A Turtle CJ Brudno JN. ASTCT Consensus Grading for Cytokine Release Syndrome and Neurologic Toxicity Associated with Immune Effector Cells. Biol Blood Marrow Transplant (2019) 25(4):625–38. 10.1016/j.bbmt.2018.12.758 PMC12180426 30592986

[14] Traube C Silver G Kearney J Patel A Atkinson TM Yoon MJ. Cornell Assessment of Pediatric Delirium: a valid, rapid, observational tool for screening delirium in the PICU*. Crit Care Med (2014) 42(3):656–63. 10.1097/CCM.0b013e3182a66b76 PMC5527829 24145848

[15] Mahadeo KM Bajwa R Abdel-Azim H Lehmann LE Duncan C Zantek N. Diagnosis, grading, and treatment recommendations for children, adolescents, and young adults with sinusoidal obstructive syndrome: an international expert position statement. Lancet Haematol (2020) 7(1):e61–72. 10.1016/S2352-3026(19)30201-7 31818728

[16] Available at: https://ctep.cancer.gov/protocoldevelopment/electronic_applications/docs/ctcae_v5_quick_reference_5x7.pdf (Accessed on January 10, 2021).

[17] Traube C Silver G Reeder RW Doyle H Hegel E Wolfe HA. Delirium in Critically Ill Children: An International Point Prevalence Study. Crit Care Med (2017) 45(4):584–90. 10.1097/CCM.0000000000002250 PMC5350030 28079605

[18] Dervan LA Di Gennaro JL Farris RWD Watson RS. Delirium in a Tertiary PICU: Risk Factors and Outcomes. Pediatr Crit Care Med (2020) 21(1):21–32. 10.1097/PCC.0000000000002126 31568239

[19] Alvarez RV Palmer C Czaja AS Peyton C Silver G Traube C. Delirium is a Common and Early Finding in Patients in the Pediatric Cardiac Intensive Care Unit. J Pediatr (2018) 195:206–12. 10.1016/j.jpeds.2017.11.064 29395177

[20] Traube C Silver G Gerber LM Kaur S Mauer EA Kerson A. Delirium and Mortality in Critically Ill Children: Epidemiology and Outcomes of Pediatric Delirium. Crit Care Med (2017) 45(5):891–8. 10.1097/CCM.0000000000002324 PMC5392157 28288026

[21] Meyburg J Dill ML Traube C Silver G von Haken R. Patterns of Postoperative Delirium in Children. Pediatr Crit Care Med (2017) 18(2):128–33. 10.1097/PCC.0000000000000993 27776085

[22] Silver G Doyle H Hegel E Kaur S Mauer EA Gerber LM. Association Between Pediatric Delirium and Quality of Life After Discharge. Crit Care Med (2020) 48(12):1829–34. 10.1097/CCM.0000000000004661 PMC8195312 33031144

[23] Silver G Kearney J Traube C Hertzig M. Delirium screening anchored in child development: The Cornell Assessment for Pediatric Delirium. Palliat Support Care (2015) 13(4):1005–11. 10.1017/S1478951514000947 PMC5031084 25127028

[24] Ista E van Beusekom B van Rosmalen J Kneyber MCJ Lemson J Brouwers A. Validation of the SOS-PD scale for assessment of pediatric delirium: a multicenter study. Crit Care (2018) 22(1):309. 10.1186/s13054-018-2238-z 30458826 PMC6247513

[25] Valdivia HR Carlin KE. Determining Interrater Reliability of the Cornell Assessment of Pediatric Delirium Screening Tool Among PIcCU Nurses. Pediatr Crit Care Med (2019) 20(4):e216–20. 10.1097/PCC.0000000000001896 30730379

[26] Winsnes K Sochacki P Eriksson C Shereck E Recht M Johnson K. Delirium in the pediatric hematology, oncology, and bone marrow transplant population. Pediatr Blood Cancer (2019) 66(6):e27640. 10.1002/pbc.27640 30697919

[27] Simeone S Rea T Gargiulo G Esposito MR Guillari A Traube C. Cornell Assessment of Pediatric Delirium: Italian cultural validation and preliminary testing. Prof Inferm (2019) 72(1):25–33. 10.7429/pi.2019.720125 31162040

[28] Barbosa M Duarte M Bastos VCS Andrade LB. Translation and cross-cultural adaptation of the Cornell Assessment of Pediatric Delirium scale for the Portuguese language. Rev Bras Ter Intens (2018) 30(2):195–200. 10.5935/0103-507X.20180033 PMC6031413 29995085

[29] He S Wang YL Zuo ZL. [Clinical application of the Chinese version of Cornell assessment of pediatric delirium: a pilot study]. Zhonghua Er Ke Za Zhi (2019) 57(5):344–9. 10.3760/cma.j.issn.0578-1310.2019.05.006 31060126

[30] Gust J Finney OC Li D Brakke HM Hicks RM Futrell RB. Glial injury in neurotoxicity after pediatric CD19-directed chimeric antigen receptor T cell therapy. Ann Neurol (2019) 86(1):42–54. 10.1002/ana.25502 31074527 PMC9375054

[31] Schreiber MP Colantuoni E Bienvenu OJ Neufeld KJ Chen KF Shanholtz C. Corticosteroids and transition to delirium in patients with acute lung injury. Crit Care Med (2014) 42(6):1480–6. 10.1097/CCM.0000000000000247 PMC4028387 24589640

[32] Harden A Puebla-Osorio N Najjar A Strati P Westin JR Nastoupil LJ. Modifiers of Endothelial Permeability in the Setting of CAR-t Therapy Related Immune Cell Associated Neurotoxicity Syndrome. Biol Blood Marrow Transplant (2020) 26(3):S258. 10.1016/j.bbmt.2019.12.452

[33] Brown ART Jindani I Melancon J Erfe R Westin J Feng L. ICU Resource Use in Critically III Patients following Chimeric Antigen Receptor T-Cell Therapy. Am J Respir Crit Care Med (2020) 202(8):1184–7. 10.1164/rccm.202002-0286LE PMC7560798 32530704

