# Supplementary figures and images for: Immune Effector Cell Associated Neurotoxicity (ICANS) in Pediatric and Young Adult Patients Following Chimeric Antigen Receptor (CAR) T-Cell Therapy: Can We Optimize Early Diagnosis?
Source: Front Oncol. 2021 Mar 8;11:634445. doi: 10.3389/fonc.2021.634445 (PMC7982581; doi:10.3389/fonc.2021.634445)

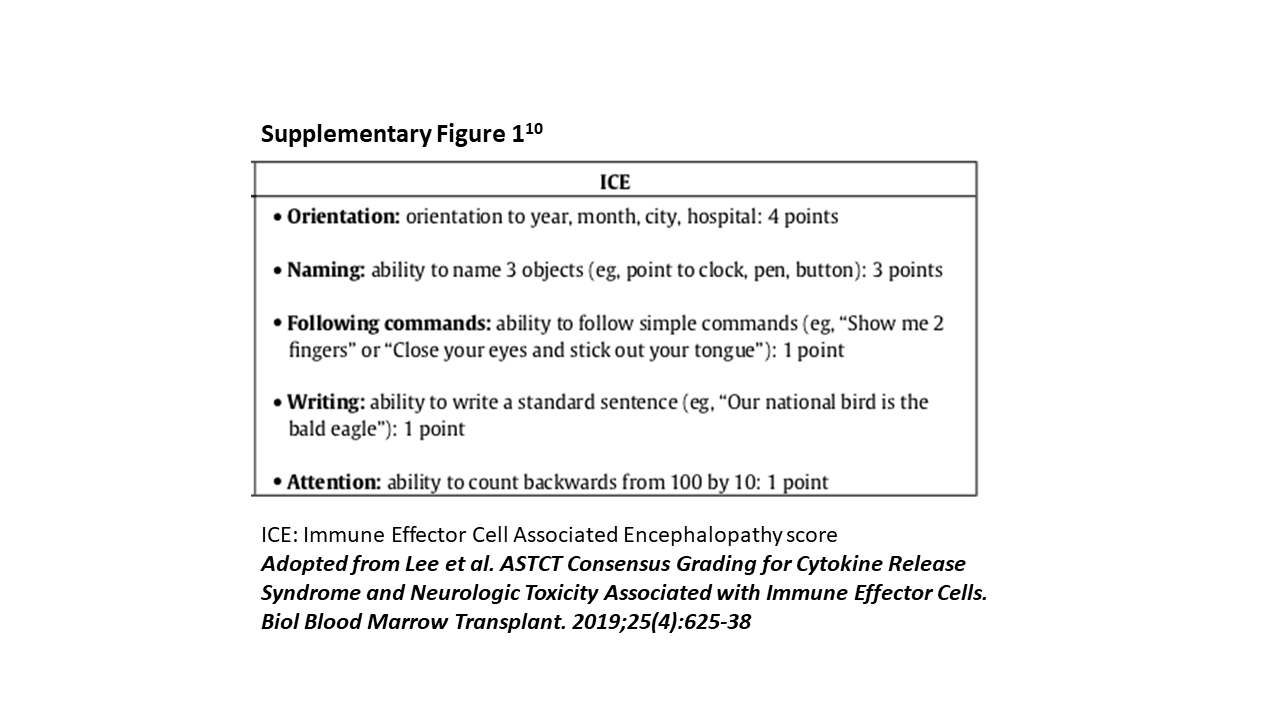

Supplement: Supplementary Figure 1 — Immune-effector cell-associated encephalopathy assessment tool for the grading of ICANS. A score of 10 represents no impairment, 7–9 grade 1 ICANS, 3–6 grade 2 ICANS, and 0–2 grade 3 ICANS. A score of 0 due to patient being unarousable and unable to perform assessment corresponds to grade 4 ICANS. [file Image_1.tif]

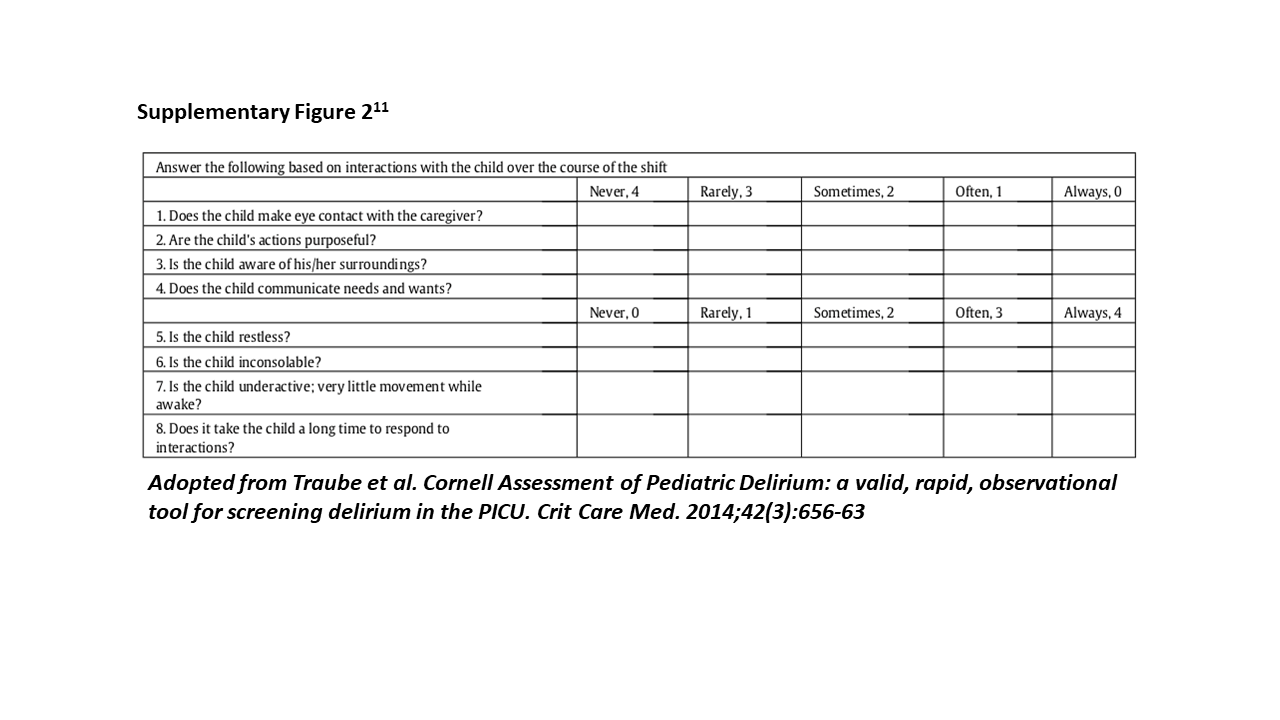

Supplement: Supplementary Figure 2 — Cornell Assessment of Pediatric Delirium. Encephalopathy assessment tool for children < 12 years of age adapted by ASTCT. Scores between 1-8 may represent no impairment, grade 1 or grade 2 ICANS and must be combined with clinical assessment. Score > 8 corresponds to grade 3 ICANS. If unable to perform CAPD due to patient being unarousable, corresponds to grade 4 ICANS. [file Image_2.tif]

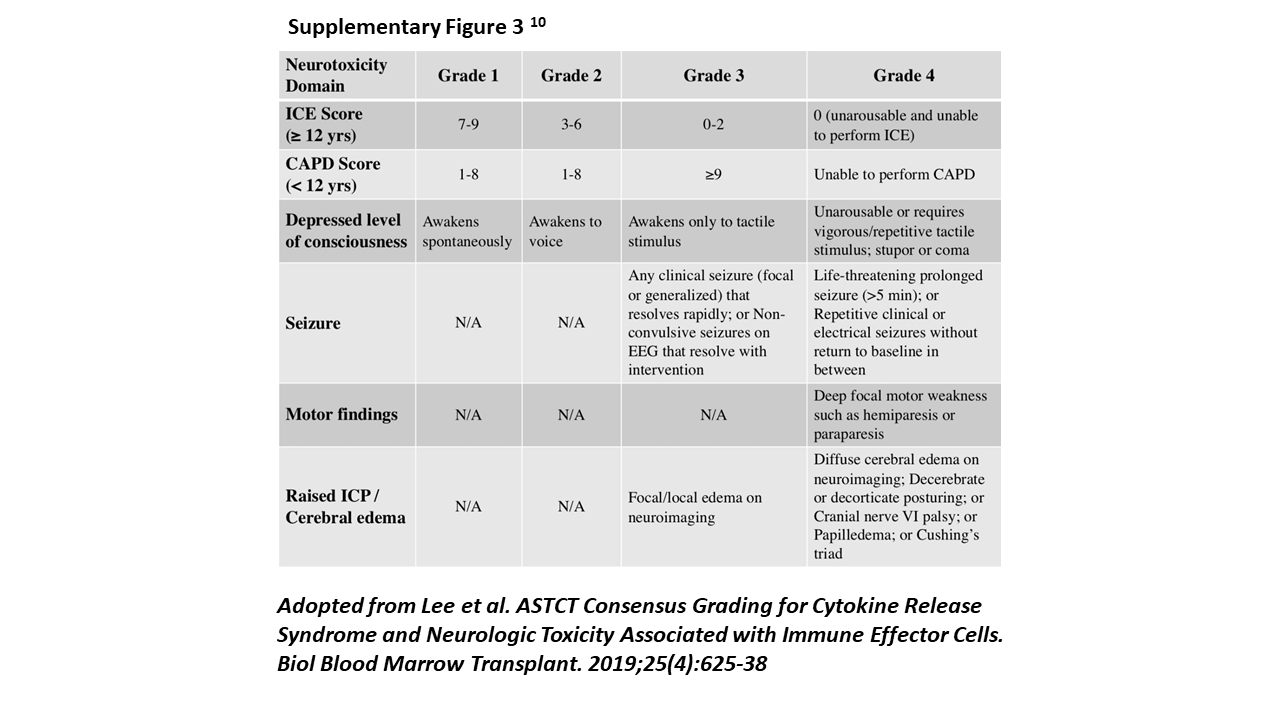

Supplement: Supplementary Figure 3 — ASTCT ICANS Consensus Grading. ICANS grade is determined by the most severe event (ICE or CAPD score, level of consciousness, seizures, motor findings, raised ICP/cerebral edema) not attributable to any other cause. [file Image_3.tif]
